# Maintaining Surgical Principles While Transitioning From Multiport to Single-port Robotic Thymectomy

**DOI:** 10.1016/j.atssr.2025.02.003

**Published:** 2025-03-04

**Authors:** Claire Perez, Lucas Weiser, Drew Bolster, Allen Razavi, Charles Fuller, Sevannah Soukiasian, Kellie Knabe, Raffaele Rocco, Harmik J. Soukiasian, Andrew R. Brownlee

**Affiliations:** 1 Division of Thoracic Surgery, Department of Surgery, Cedars-Sinai Medical Center, Los Angeles, California

## Abstract

Thymectomy is a treatment option for new-onset or treatment-resistant myasthenia gravis in patients with muscle-type acetylcholine receptor autoantibodies, and it is the standard treatment of early-stage thymomas. However, patients with severe forms, particularly those with titin and ryanodine receptor antibodies, may require long-term immunosuppressive therapy instead. The optimal approach to thymectomy remains debated, with minimally invasive techniques like video-assisted and robot-assisted thoracoscopic surgery offering better perioperative outcomes. Single-port robot-assisted thymectomy has emerged as a safe alternative, allowing a single subxiphoid incision and affording the inherent benefits of the robotic platform. This study describes our approach to single-port robot-assisted thymectomy using a subxiphoid incision.

Thymectomy is a treatment option for new-onset myasthenia gravis in patients with muscle-type acetylcholine receptor autoantibodies and the standard treatment of early-stage thymomas.^1^^,^^2^ In myasthenic patients, disease-related limitations in pulmonary mechanics and postoperative pain and inflammatory response can slow recovery and result in disease flares. This has led to a growing interest in refining the indications for surgery, with an emphasis on reducing the burden of recovery. The optimal approach and extent of thymectomy are still debated, especially with the growing adoption of minimally invasive techniques.^3^

A study using data from The Society of Thoracic Surgeons General Thoracic Surgery Database found that minimally invasive thymectomy methods, such as video-assisted thoracoscopic surgery (VATS) and robot-assisted thoracoscopic surgery, resulted in better perioperative outcomes compared with traditional transthoracic and transcervical approaches.^4^ This trend may be further enhanced by technologic advancements, including the development of single-port robot-assisted surgery. Studies have demonstrated that single-port robot-assisted thoracic surgery is both feasible and safe, with minimal conversion to VATS and a low incidence of postoperative complications.^5^ Whereas the clinical benefits of and indications for single-port thymectomy remain undetermined, a multi-institutional, propensity score–matched study revealed that single-port procedures have a lower conversion rate to multiport approaches compared with single-port VATS.^6^ In this study, we describe our approach to a single-port robot-assisted thymectomy using a subxiphoid incision.

This case involves a 30-year-old woman with a medical history of acetylcholinesterase antibody-positive myasthenia gravis, first diagnosed in 2019. She initially presented with symptoms of dysphonia, dysphagia, and muscle weakness, which improved with medical treatment and immunosuppression. However, her symptoms relapsed in 2020, leading to a referral for thymectomy. After an extensive discussion of the risks and benefits of single-port vs multiport robot-assisted thymectomy, the patient elected to proceed with a subxiphoid single-port robot-assisted thymectomy.

## Technique

**Figure 1:**
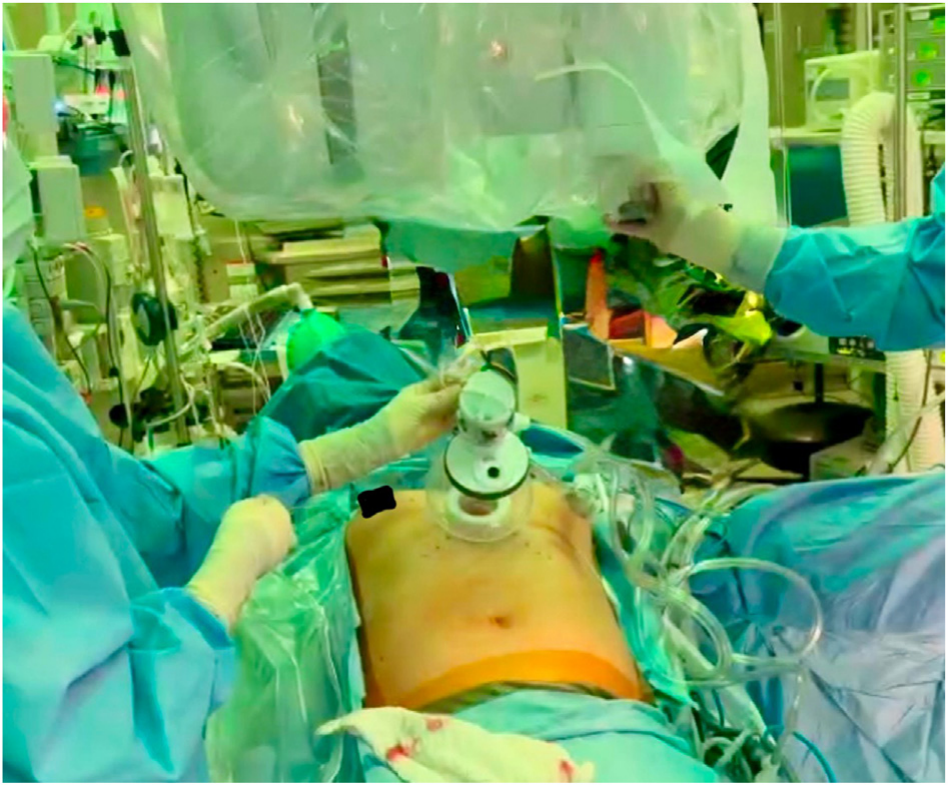
Subxiphoid incision with robot docked.

The patient is positioned supine with a roll placed transversely at the level of the xiphoid process. A double-lumen tube is used for intubation in case 1-lung ventilation is needed to provide adequate space and visualization, although we have found that low tidal volumes and insufflation often mitigate this need. A 5-cm incision is made approximately 1 fingerbreadth below the xiphoid process (Figure 1). Dissection is carried through the subcutaneous tissue and extended cranially and retrosternally. Blunt finger dissection is used to create the retrosternal space and to access the bilateral pleura. Careful finger dissection should be performed to prevent any violation of the peritoneum (Video). The camera is introduced to confirm sufficient dissection. A single-port wound protector is then placed, and the robotic system is docked. Insufflation is set to 10 mm Hg. The procedure uses a 4-arm robotic approach: a camera, a fenestrated bipolar device in the left hand, a round-tooth forceps from below, and a monopolar cautery in the right hand using low energy.

**Figure 2:**
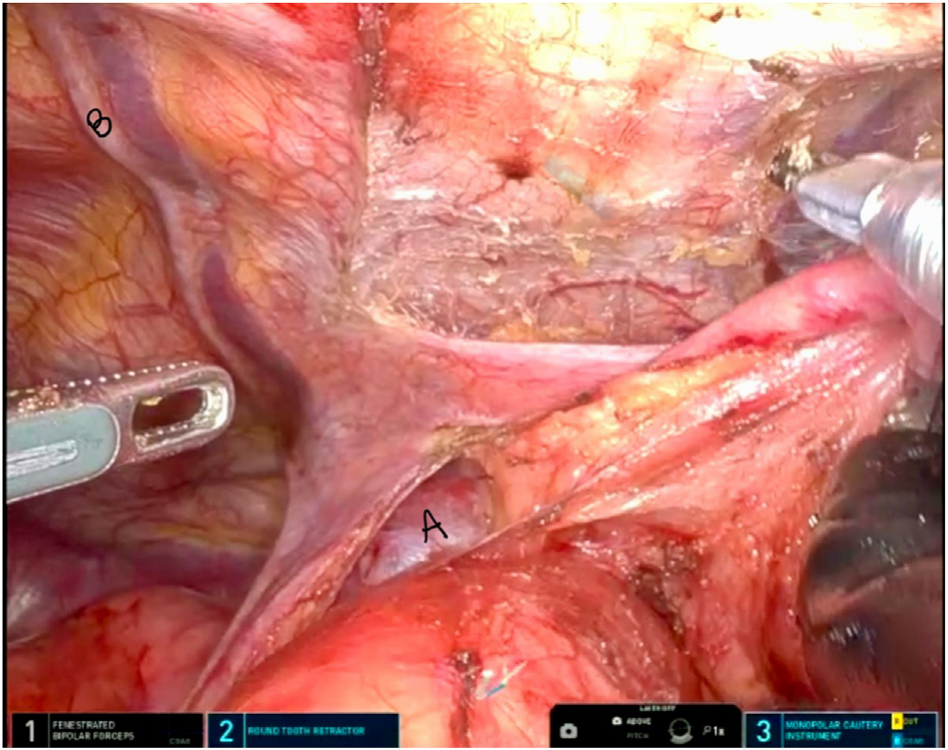
Clear visualization of the innominate vein (**A**) and right phrenic nerve (**B**).

The procedure begins by opening the bilateral mediastinal pleura to access the thoracic cavity. It is crucial to conduct a brief finger dissection at the caudal aspect of the sternum to create sufficient space for the camera and articulating arms to be fully positioned in the chest. The most inferior portion of the thymus can also be mobilized before docking the robot. The thymus is then carefully separated from the chest wall and pericardium by a combination of monopolar or bipolar (Maryland bipolar) cautery and blunt dissection to mobilize its inferior portion. The dissection proceeds cranially, with close attention to identifying and preserving both the left and right phrenic nerves (Figure 2). When the monopolar cautery is used, it is important to use a low energy of 4 to avoid thermal injury to the phrenic nerves.

The subxiphoid approach offers a clear bilateral view of the phrenic nerves, which is particularly critical in patients with myasthenia gravis, ensuring the complete exenteration of thymic tissue while preserving diaphragmatic function. This approach also allows optimal visualization of the vascular structures encountered during the cranial dissection. At times, we reposition our instruments, placing the fenestrated bipolar device in the left hand for more precise dissection around the innominate vein. The ports work around a single fulcrum, making the instrument exchanges crucial for good exposure. Clips are applied to venous structures before transection. The Maryland bipolar is used for delicate dissection near the innominate vein. Intraoperative bleeding is most likely to occur during this portion from the venous branches of the innominate vein; pressure may be applied with a cigar or the application of hemostatic agents to prevent conversion to median sternotomy. A flexible tip suction catheter, which can be positioned by the surgeon, is also used.

Once the thymus is freed from its venous attachments, attention shifts to mobilizing the left thymic horn by bipolar cautery for finer dissection. Retraction of the thymus caudally to the right is key at this stage to enhance visualization. After the thymus is fully detached, Surgicel is applied to ensure hemostasis. Finally, a 24F Blake drain is placed through the subxiphoid incision.

## Comment

Subxiphoid single-port robot-assisted thymectomy is a safe and feasible procedure that provides excellent visualization of both phrenic nerves, which is an advantage not typically seen with the traditional transthoracic approach. This technique also offers the benefit of a more aesthetically pleasing single incision and reduced postoperative pain as it avoids the intercostal nerves. Previous studies have demonstrated that the subxiphoid approach is less invasive than the lateral intercostal approach, even when a multiport system is used.^7^ Although a higher body mass index presents challenges related to retraction and maneuverability,^8^ our practice has not encountered significant difficulties in patients with elevated body mass index once appropriate access is obtained. This approach also allows good visualization, even for thymoma resection, although we typically limit tumor size to <5 cm. Overall, the subxiphoid robot-assisted technique represents a less invasive and practical option for thymectomy.
